# Multiscale Assessment of Nanoscale Manufacturing Process on the Freeform Copper Surface

**DOI:** 10.3390/ma13143135

**Published:** 2020-07-14

**Authors:** Yafei Xu, Handing Liu, Liuyang Zhang, Matthew Becton

**Affiliations:** 1 State Key Laboratory for Manufacturing Systems Engineering, Jiaotong University, Xi’an 710049, China; xyf2492229210@stu.xjtu.edu.cn (Y.X.); lhd.1992.lc@stu.xjtu.edu.cn (H.L.); 2 Xi’an Jiaotong University Shenzhen Academy, Nanshan District, Science and Technology Park, Shenzhen 518057, China; 3 College of Engineering, University of Georgia, Athens, GA 30602, USA; becton@uga.edu

**Keywords:** multiscale simulation, quasi-continuous method, nanocutting, copper sample, cutting tool parameters

## Abstract

The nanocutting has been paid great attention in ultra-precision machining and high sealing mechanical devices due to its nanometer level machining accuracy and surface quality. However, the conventional methods applicable to reproduce the cutting process numerically such as finite element (FE) and molecular dynamics (MD) are challenging to unveil the cutting machining mechanism of the nanocutting due to the limitation of the simulation scale and computational cost. Here a modified quasi-continuous method (QC) is employed to analyze the dynamic nanocutting behavior (below 10 nm) of the copper sample. After preliminary validation of the effectiveness via the wave propagation on the copper ribbon, we have assessed the effects of cutting tool parameters and back-engagement on the cutting force, stress distribution and surface metamorphic layer depth during the nanocutting process of the copper sample. The cutting force and depth of the surface metamorphic layer is susceptible to the back-engagement, and well tolerant to the cutting tool parameters such as the tool rank angle and tool rounded edge diameter. The results obtained by the QC method are comparable to those from the MD method, which indicate the effectiveness and applicability of the modified QC method in the nanocutting process. Overall, our work provides an applicable and efficient strategy to investigate the nanocutting machining mechanism of the large-scale workpiece and shed light on its applications in the super-precision and high surface quality devices.

## 1. Introduction

With the rapid development of ultra-precision machining technology in recent decades, the nanocutting that offers nanometer level machining accuracy and surface quality has been an optimal approach to fulfill the fabrication of the micro/nanostructures such as the precision optical devices and high sealing mechanical devices [1,2,3]. In the nanocutting machining process, some phenomena that are related to the cutting machining mechanism are hard to be observed by experiment due to the limitations of complexity and minimization of nanocutting process, which limits the optimal tuning of the nanocutting process. However, numerical simulation methods such as the finite element and molecular dynamics might provide an alternative way to understand the internal cutting machining mechanism, especially the nanoscale interaction mechanism between the tool and workpieces during the nanocutting process.

The finite element (FE) method based on continuum mechanics that ignores the interaction between the atoms and bonds of the workpiece is a conventional approach to investigate the deformation and mechanical properties during the cutting process [4,5]. The continuum and homogeneity are normally required to fulfill the necessary conditions for the appropriate FEM implementation. At present, it has been employed extensively to predict cutting machining conditions such as the deformation, cutting force, stress distribution and temperature distribution during the cutting process in the macro domain [6,7,8,9]. However, for the machining accuracy down to the nanoscale, FE simulation results might be compromised even if the calculation accuracy can be improved by mesh refinement [10], which is attributed to the large errors of the constitutive equations at the nanoscale [11]. Therefore, the FE method is an inappropriate choice to unveil the details during the nanocutting process.

Since the MD method is first proposed to be applied in the machining by Belak in the late 1980s [12], it has been another extensively used approach to investigate the deformation and mechanical properties during the cutting process [13,14,15]. In the MD method, the simulation model is normally established based on the interatomic and intra-atomic interactions between the atoms and bonds of the workpiece that can be described by proper potential functions at the atomic scale, which indicates the micro deformation of the model with high accuracy. Due to the atomic level time and length resolution, the MD method has been extensively applied in the nanocutting process. Previously, Lai et al. investigated the surface topography, cutting force and subsurface deformation in partially overlapped nanocutting of monocrystalline germanium under different feeds conditions by the MD method [16]. Wang et al. indicated that structural distribution the subsurface defects and its evolution by analyzing the dislocation evolution and atomic migration during the nanocutting process of the copper sample [14]. However, due to the limitation of the computational resources, the MD method is often confined to the length range with few nanometers, which limits the applications of the MD method in the large scale manufacturing process on the large size of workpiece.

To balance the simulation accuracy and the computational efficiency, multiscale simulation methods that combine the FE method and MD method have been developed and applied extensively in the field of computational mechanics. For example, the FEAt method that adopts the transition regions to directly couple the atomic region and continuous region has been successfully applied in some crystals with simple structure such as body-centered cubic (bcc) crystals [17]. The macro-atomistic and ab-initio dynamics method (MAAD) that was proposed by Abraham et al. [18] introduces the tight-binding model based on the FEAt, which is mainly applied in crystal crack propagation. The coarse grained molecular dynamics (CGMD) based on a statistical coarse graining approximation employs a simplified energy formula to reduce the computational cost, which is only suitable for the special situations where energy can be simplified [19]. Comparing to the above multiscale methods, the quasi-continuum (QC) method that does not directly couple the atomic region and the continuous region by using inaccurate theoretical methods, but integrates the finite element theory into the molecular calculation, and makes the simulation results closer to the real physical conditions, which has been extensively applied in many aspects such as nanoindentation [20,21], structural failure [22,23], microdefect [24], crack propagation [25,26] and soon. However, few work has been carried out to apply the QC method in the investigation of the nanocutting process, although such a simulation method emerges great potential to solve the multiscale nanocutting machining process with multiplicity and complexity [10,27].

In the work, a multiscale simulation method based on the QC method is employed to explore the effects of the cutting tool parameters and back-engagement on the cutting force, stress distribution and depth of the surface metamorphic layer by establishing the effective multiscale cutting model during the nanocutting process of copper sample. Additionally, since it is difficult to realize the nanocutting experiment below 10 nm, the MD method with high simulation accuracy is utilized to validate the effectiveness and applicability of the modified QC method under the same cutting conditions during the nanocutting machining process. Our work might provide an applicable and efficient computational strategy to investigate the nanocutting machining mechanism of the workpiece.

## 2. Method and Simulation Model

In this section, we first modified the traditional QC method by introducing the Cauchy local stress and additional material removal strategy to simulate and characterize the actual nanocutting process accurately. Then, a multiscale nanocutting model of copper sample is established by the modified QC method to investigate the nanocutting process and machining mechanism under different cutting conditions, which is verified by the MD model.

### 2.1. Modified QC Method

QC method, a multiscale simulation method that integrates continuum mechanics and discrete atoms was first proposed by Tadmor et al. in 1996 [28]. The method employs automatic mesh generation to refine the dislocation core region to the atomic scale, while a relatively rough description is conducted by the “representative atoms” following the finite element theory for the region away from the dislocation core region. To reduce the computational cost, the Cauchy–Born rule that assumes the continuum energy density can be obtained by the partial atomic potential with the uniform deformation gradient is employed to simplify the QC method. According to the Cauchy–Born rule, the continuum first Piola–Kirchhoff stress tensor based on the finite element theory can elegantly simulate the stress distribution in the continuum domain, while it will be limited in the dislocation core region due to the limitation of length scale. Here, the Cauchy local stress in an atomic system defined by Hardy [29] was employed to accurately calculate the stress distribution in the dislocation core regions due to its independence of length scale and localization function than the Piola–Kirchhoff stress (Supplementary Figure S1). In the Cauchy stress calculation, the continuum fields such as the mass density ρ, linear momentum density p and internal energy density e are defined by using the following localization function Ψ as [29,30]:

(1) $$\rho \left(x,t\right)=\sum_{\alpha =1}^{N}m^{\alpha }\psi \left(x^{\alpha }-x\right)$$

(2) $$p\left(x,t\right)=\sum_{\alpha =1}^{N}m^{\alpha }\upsilon^{\alpha }\psi \left(x^{\alpha }-x\right)$$

(3) $$e\left(x,t\right)=\sum_{\alpha =1}^{N}\left\{\frac{1}{2}m^{\alpha }\left(\upsilon^{\alpha 2}\right)+\phi^{\alpha }\right\}\psi \left(x^{\alpha }-x\right)$$

where x and υ represent the atomic position and velocity, respectively; t represents the simulation time and $m^{\alpha }$ and $\phi^{\alpha }$ represent the atomic mass and potential energy, respectively. The localization function ψ allows each atom to contribute to a continuum property at the position x at time t with a normalized function. A bond function $B^{\alpha \beta }\left(x\right)$ that represents a weighted fraction of the bond length segment between atoms α and β within the characteristic volume can be expressed as [31]:

(4) $$B^{\alpha \beta }\left(x\right)=\int_{0}^{1}\psi \left(\lambda x^{\alpha \beta }+x^{\beta }-x\right)d\lambda$$

Based on the above definitions and the basic assumptions about the energy and force for the atoms from reference [32], the local stress at the position x at time t can be expressed as:

(5) $$\sigma \left(x,t\right)=-\left\{\frac{1}{2}\sum_{\alpha =1}^{N}\sum_{\beta \neq \alpha }^{N}x^{\alpha \beta }⊗F^{\alpha \beta }B^{\alpha \beta }\left(x\right)+\sum_{\alpha =1}^{N}m^{\alpha }u^{\alpha }⊗u^{\alpha }\psi \left(x^{\alpha }-x\right)\right\}$$

where $u^{\alpha }=\upsilon^{\alpha }-\upsilon$. The local stress expression is robust to the atomic system and the dynamic continuum. Especially, when the characteristic volume is taken to be the entire volume of the atomic system, the average Cauchy stress within the system can be obtained by [33,34]:

(6) $$\overset{-}{\sigma }=\left(1/V\right)\int_{R^{3}}\sigma \left(x\right)d^{3}x$$

Note that the QC method is a two-dimensional simulation method, the volume element V should be calculated by the circular microelement instead of the spherical microelement. Therefore, Equation (6) can be simplified as:

(7) $$\overset{-}{\sigma }=\left(1/V\right)\int_{R^{2}}\sigma \left(x\right)d^{2}x$$

Here, the unit of the obtained stress is $ev/\overset{o}{A}$, and thus the local stress in the macroscopic domain is obtained by dividing the lattice thickness.

Additionally, since the pristine QC method lacks an unrealistic material removal module that limits the applications of QC method in the nanocutting process, an effective material removal strategy was introduced to fulfill the chip formation and removal under large deformation conditions. The core of material removal is to deal with a series of problems caused by the unit removal in the cutting process, such as destruction of the initial meshes, the dimension error of the information storage matrix and so on. Here, we additionally calculated the side length of each unit in the atomic region at each load step. When the side length of the unit that locates at the edge of the model exceeds the cutoff range of interaction between workpiece atoms, the corresponding deformation units will be automatically removed [35]. Then, the representative atom region and information storage matrix related to the deleted units are updated at the current load step to maintain the effectiveness of the QC method after material removal (Supplementary Figure S2).

### 2.2. Model Setup

**Figure 1:**
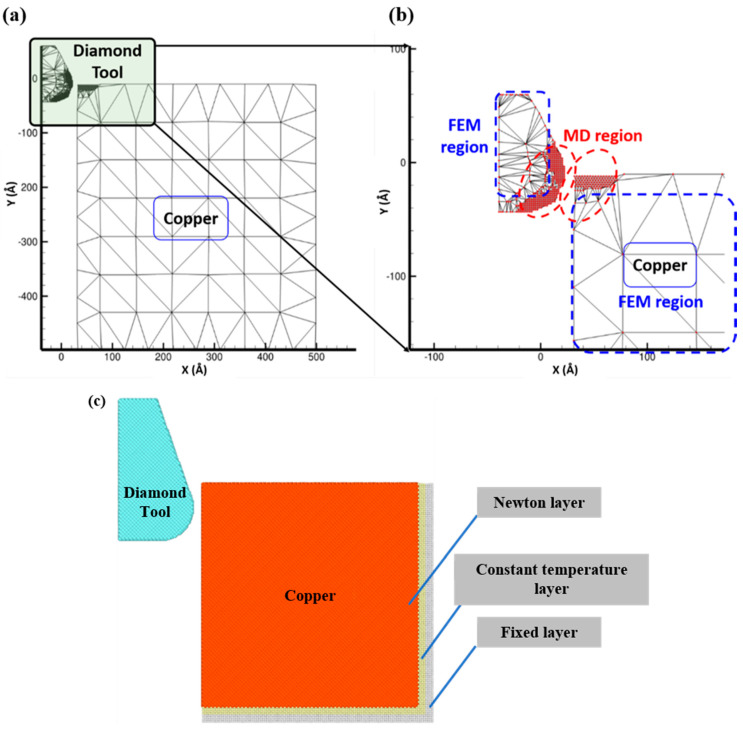
(**a**) The schematic diagram of multiscale cutting model for the nanocutting process of the copper sample; (**b**) zoom-in view of the interfacial contact between the diamond machining tool and the copper sample and (**c**) the schematic diagram of the MD cutting model for the nanocutting process of the copper sample.

In the work, we employed a multiscale nano-metric cutting model of copper sample with the bottom side fixed to investigate the nanocutting process and machining mechanism under different cutting conditions by the modified QC method. The schematic illustration of the multiscale cutting model is shown in Figure 1a. In the multiscale model, the diamond tool is simplified as a rigid body to cut the free surface of copper sample at a constant speed of 1$\overset{\circ }{A}$/ps along the x axis. The interatomic interaction in the copper sample is defined by the Embedded-Atom Method (EAM) potentials with detailed parameters (Supplementary Table S1). The contact region between the diamond tool and the copper sample is modeled as the atomistic region (MD region) while the remaining region is modeled as the finite element method region (FEM region), as shown in Figure 1b. The initial size of the multiscale cutting model is set as 500$\overset{\circ }{A}$×500$\overset{\circ }{A}$ with approximately 510 representative atoms, which is much less than 250,000 atoms included in the MD method, and thus saves computational time. Additionally, to validate the applicability of the modified QC method in the nanocutting process, the MD model of copper sample with the fcc lattice is also established by the EAM potentials with the same cutting parameters as shown in Figure 1c. The dimensions of the MD model are 500$\overset{\circ }{A}$×500$\overset{\circ }{A}$×4a, where a is the lattice constant of the copper (3.62$\overset{\circ }{A}$).

## 3. Results and Discussion

In this section, we first validated the effectiveness and applicability of the multiscale QC method in coupling the discrete MD region and the continuous FEM region of the multiscale model of the crystal copper sample. To better understand the cutting process and machining mechanism during the nanocutting process, we then investigated the effects of the back-engagement, tool rank angle and tool rounded edge diameter on the cutting force, stress distribution and depth of surface metamorphic layer by the modified QC method, respectively, which were further verified by the MD method.

### 3.1. Model Validation

**Figure 2:**
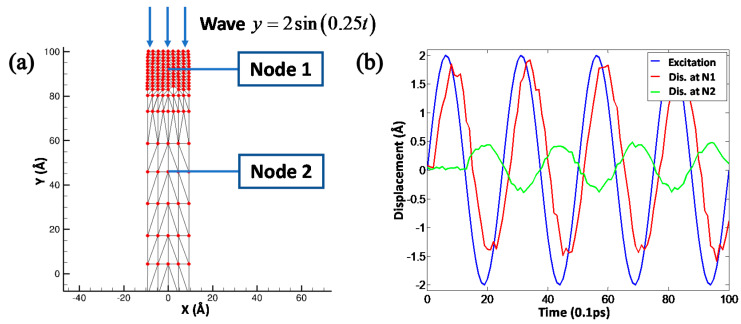
The validation of the QC method by 1-D wave propagation. (**a**) A sinusoidal actuation is imposed on the terminal of copper ribbon. (**b**) The displacement of nodes 1 and 2 with respect to the simulation time.

Initially, we investigated the propagation characteristics of the 1-D wave in the copper ribbon to validate the effectiveness and accuracy of the QC method in the coupling finite element region and atomic region, especially the wave propagation across the interface with a mismatching length scale and time scale. As shown in Figure 2a, a sinusoidal actuation is imposed on the terminal of the copper ribbon with the actuation amplitude 2$\overset{\circ }{A}$. The displacement at node 1 in the MD region and node 2 in the FEM region were recorded to monitor the mechanical wave propagation from the MD region to the FEM region. Figure 2b illustrates the displacement of nodes 1 and 2 with respect to the simulation time. It can be found that node 1 in the MD region exhibited a vibrational behavior closer to the initial actuation but with a small phase delay than node 2. The attenuated displacement at node 2 indicates the mechanical wave experienced severe energy dissipation in the propagation pathway. To further validate the effectiveness of the multiscale model quantitatively, the vibrational displacement of nodes 1 and 2 were fitted sinusoidally. It can be clearly found that the vibrational periods of nodes 1 and 2 were consistent with the initial actuation period of 0.25, however the phase delay that represents the time of wave propagation from the initial terminal to two nodes was 0.2161 ps and 1.2682 ps, respectively. Therefore, the average wave speed through the copper ribbon could be estimated as 4301.77 m/s and 4242.23 m/s at nodes 1 and 2, respectively, which also were similar to the physical wave speed constant of 3750 m/s. It can be concluded that multiscale QC method can effectively couple the discrete MD region and the continuous FEM region to simulate the multiscale cutting process of the crystal copper sample.

In the following part, to better understand the cutting process and machining mechanism during the nanocutting process, the effects of the different cutting and tool parameters such as back-engagement, tool rank angle and tool rounded edge diameter on the cutting force, stress distribution and depth of surface metamorphic layer were investigated by the modified QC method.

### 3.2. Effect of the Back-Engagement

**Figure 3:**
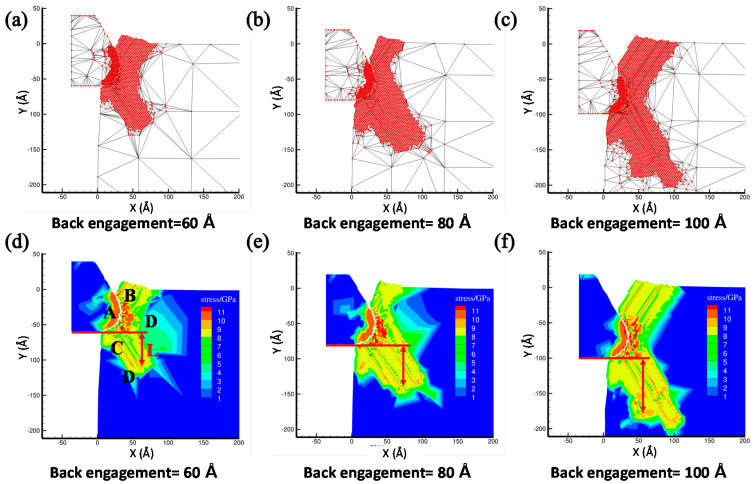
The atomic displacement and corresponding stress distribution at the 200th step with varying the back-engagement from 60 to 100 Å. (**a**) The atomic displacement when back-engagement = 60 Å; (**b**) the atomic displacement when back-engagement = 80 Å; (**c**) the atomic displacement when back-engagement = 100 Å; (**d**) the corresponding stress distribution when back-engagement = 60 Å; (**e**) the corresponding stress distribution when back-engagement = 80 Å and (**f**) the corresponding stress distribution when back-engagement = 100 Å.

The critical effect of the back-engagement on cutting machining was investigated by the modified QC method. With the initial tool rank angle 30° and rounded edge diameter 60 Å, Figure 3a–c illustrates the atomic displacement during the cutting process of the copper sample at the 200th load step by varying the back-engagement from 60 to 100 Å. It can be found that the lattices at the contact region between the diamond tool and the copper sample underwent the sequential deformation and mesh refinement when the tool moved towards the workpiece (as shown in Figure 3a). Due to the compression, the atoms in the deformation region will be rearranged when the strain energy reserved in the deformed lattice exceeds the interatomic interaction of the copper atoms. Additionally, the dislocation band that is composed of the upward dislocation band and downward dislocation band will be generated in the deformed lattices to release the strain energy when the primary shear deformation caused by the intrusion of the cutting tool is larger than the critical lattice of the copper sample. The upward dislocation band accumulated along the tool rake face and gradually form a chip hill, and the downward dislocation band accumulates along the lower left side of cutting tool and flies back to the sample to form the machined surface. With the back-engagement increases, the dislocation band was aggravated and the chip thickened gradually during the nanocutting process of the copper sample. The results obtained are consistent with previous work [36,37].

**Figure 4:**
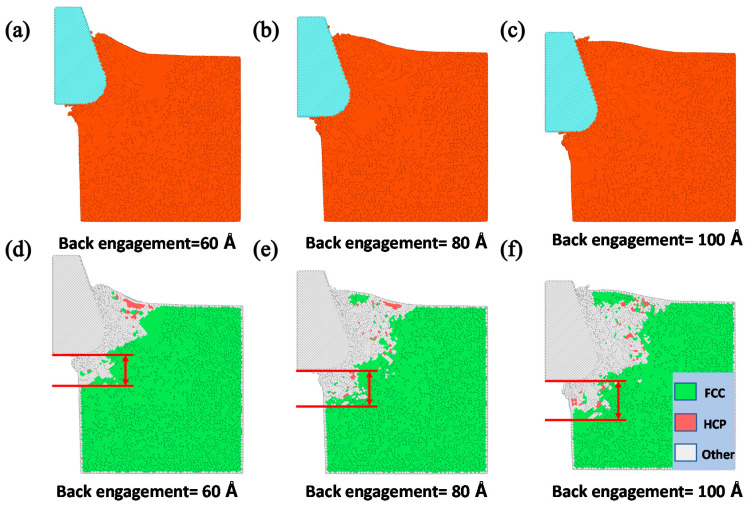
The atomic displacement and corresponding lattice distribution in MD simulation at the 200th load step with varying back-engagement from 60 to 100 Å. (**a**) The atomic displacement when back-engagement = 60 Å; (**b**) the atomic displacement when back-engagement = 80 Å; (**c**) the atomic displacement when back-engagement = 100 Å; (**d**) the corresponding lattice distribution when back-engagement = 60 Å; (**e**) the corresponding lattice distribution when back-engagement = 80 Å and (**f**) the corresponding lattice distribution when back-engagement = 100 Å.

To further unveil the underlying cutting deformation mechanism of the copper sample under different back-engagement, the corresponding stress distribution that indicates the cutting conditions of the workpiece was analyzed to elaborate the effect on the dislocation band and the surface metamorphic layer during the cutting process. The depth of the surface metamorphic layer *L* that represents the maximum depth of atoms up to the critical stress threshold can be obtained from the stress distribution, which greatly affected the surface machining quality of the workpiece. Figure 3d–f illustrates the corresponding stress distribution and depth of the surface metamorphic layer at the 200th step with the back-engagement from 60 to 100 Å. As shown in Figure 3d, when the back-engagement was set to 60 Å, the stress distribution mainly concentrated in the contact region A due to the intrusive compression, which resulted in the stress concentration and drastic deformation. Consequently, it is observed that large stress was also accumulated in the region B and C along the dislocation band while small stress concentration was distributed in region D away from the deformation region, which led to the formation of the chip and surface metamorphic layer. With the back-engagement increases shown in Figure 3, the stress distribution, the dislocation band and the depth of surface metamorphic layer increased and expanded gradually. Additionally, Figure 4 illustrates the atomic displacement and corresponding lattice distribution in MD simulation, and indicates that the depth of HCP lattice that represents the damaged atoms at the contact region increased gradually with the increment of back-engagement, which were consistent with the above results obtained by the QC method.

**Figure 5:**
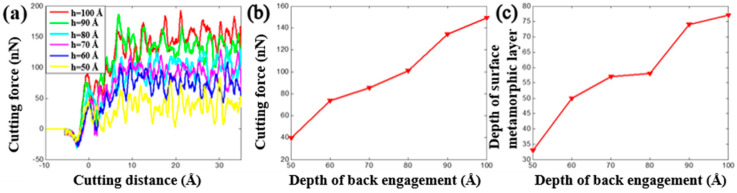
The cutting force and depth of surface metamorphic layer obtained from QC by varying the depth of back-engagement. (**a**) Cutting force vs. the cutting distance under different back-engagement h; (**b**) evolution of the average cutting force when varying the depth of back-engagement and (**c**) evolution of the depth of the surface metamorphic layer when varying the depth of back-engagement.

**Figure 6:**
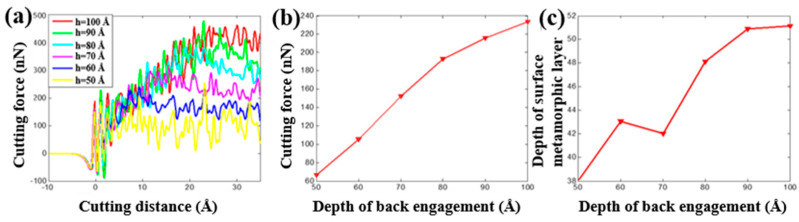
The cutting force and depth of metamorphic layer obtained from MD by varying the depth of back-engagement. (**a**) Cutting force vs. the cutting distance under different back-engagement h; (**b**) evolution of the average cutting force when varying the depth of back-engagement and (**c**) evolution of the depth of the surface metamorphic layer when varying the depth of back-engagement.

It is worth mentioning that the cutting force is an important factor to affect the nanocutting process. Figure 5 and Figure 6 show the evolution of the cutting force and depth of the surface metamorphic layer obtained by the QC and MD method under different back-engagement, respectively. It can be found that the cutting force increased sharply with the increment of cutting distance when the tool cut into the workpiece for 10 Å, and then gradually stabilized as shown in Figure 5a and Figure 6a. During the cutting process, the cutting force underwent reasonable fluctuations that were attributed to the evolution of the dislocation band. Figure 5b illustrates the average cutting force obtained by the QC method from 25 to 35 Å cutting distance increased approximately linearly from 40 to 150 nN with the increment of back-engagement, while the average cutting force obtained by the MD method increased from 60 to 240 nN (as shown in Figure 6b). Similarly, the depth of surface metamorphic layer was so. Overall, even though the cutting force amplitudes were slightly mismatched due to the deficiency of the multiscale QC method, the cutting force and the depth of the surface metamorphic layer from the QC and MD method mentioned above had a similar trend, which validated the applicability of the modified QC method to unveil the effect of the back-engagement on the cutting machining mechanism in the nanocutting process.

### 3.3. Effect of the Tool Rake Angle

**Figure 7:**
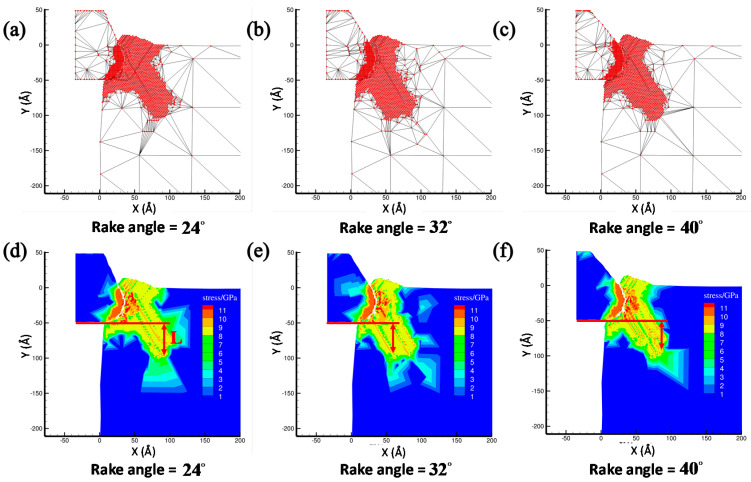
The atomic displacement and corresponding stress distribution at the 200th step by varying the tool rank angle from 24 to 40°. (**a**) The atomic displacement when the rake angle = 24°; (**b**) the atomic displacement when the rake angle = 32°; (**c**) the atomic displacement when the rake angle = 40°; (**d**) the corresponding stress distribution when the rake angle = 24°; (**e**) the corresponding stress distribution when the rake angle = 32° and (**f**) the corresponding stress distribution when the rake angle = 40°.

**Figure 8:**
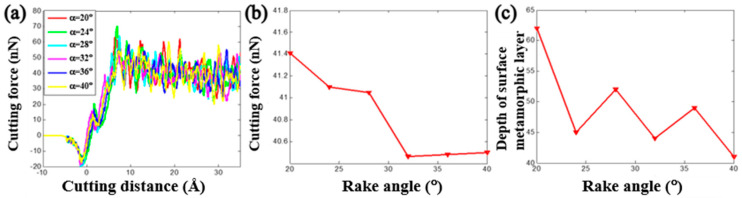
The cutting force and depth of the surface metamorphic layer obtained from QC by varying the tool rake angle. (**a**) Cutting force vs. the cutting distance under different tool rake angle α; (**b**) evolution of the average cutting force when varying the tool rake angle and (**c**) evolution of the depth of the surface metamorphic layer when varying the tool rake angle.

**Figure 9:**
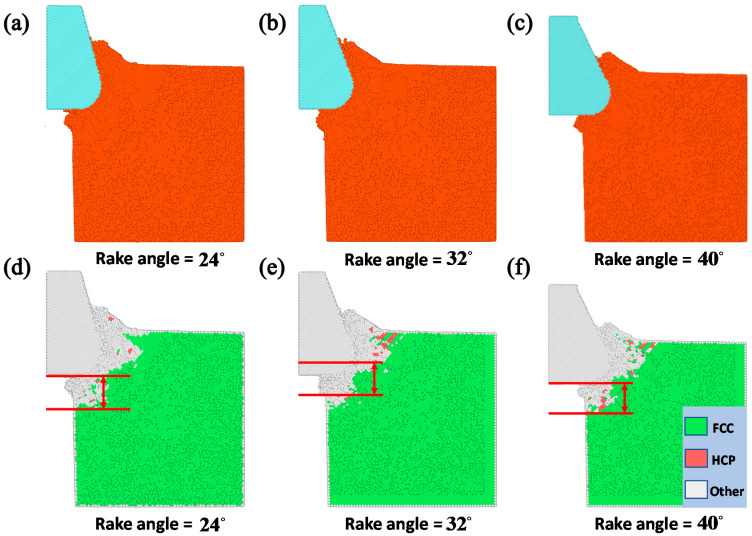
The atomic displacement and corresponding lattice distribution in the MD simulation at 200th step by varying the tool rake angle. (**a**) The atomic displacement when the rake angle = 24°; (**b**) the atomic displacement when the rake angle = 32°; (**c**) the atomic displacement when the rake angle = 40°; (**d**) the corresponding lattice distribution when the rake angle = 24°; (**e**) the corresponding lattice distribution when the rake angle = 32° and (**f**) the corresponding lattice distribution when the rake angle = 40°.

**Figure 10:**
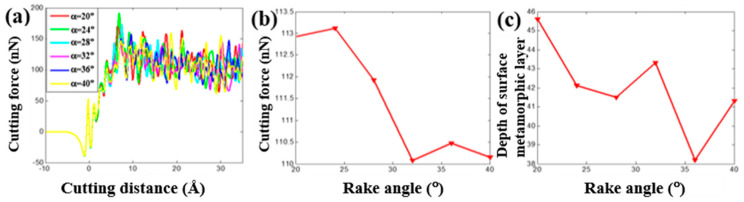
The cutting force and depth of the surface metamorphic layer obtained from MD by varying the tool rake angle. (**a**) Cutting force vs. the cutting distance under different tool rake angles α; (**b**) evolution of the average cutting force when varying the tool rake angle and (**c**) evolution of the depth of the surface metamorphic layer when varying the tool rake angle.

The tool rank angle is one important cutting tool parameter that needs to be taken into account for investigating the cutting process of the copper sample. By fixing the initial back-engagement 50 Å and tool rounded edge diameter 60 Å, Figure 7a–c illustrates the atomic displacement variation during the cutting process of the copper sample at the 200th load step when varying the tool rank angle from 24 to 40°. It can be found that the deformation of the lattice mainly located on the contact region between the diamond tool and the copper sample, and the chip thickness slightly increased with the increment of the tool rake angle, which can be verified by the internal stress distribution of the workpiece in Figure 7d–f where the stress concentrated on the contact region and dislocation band. Figure 8a illustrates that the cutting force varied with cutting distance under a different tool rake angle. It can be found that the cutting force initially increased sharply with the increment of the cutting distance, and then gradually stabilized, which followed a similar trend to the effect of the back-engagement (Figure 5a). Figure 8b illustrates the variation of the average cutting force from 25 to 35 Å cutting distance with respect to the tool rake angle. It can be observed that the average cutting force decreased slightly from 41.1 to 40.2 nN with the increment of the tool rake angle, which can be attributed to the decrement of the friction between the front surface of the tool and the workpiece. The depth of the surface metamorphic layer exhibited a decreasing trend with some fluctuation with the increment of the tool rake angle (as shown in Figure 8c). Additionally, the results obtained by MD simulation are shown in Figure 9 and Figure 10, indicating that the cutting force and the depth of HCP lattice decreased gradually with the increment of the tool rank angle, which are comparable to the above results obtained by the QC method. Overall, It concludes that the small tool rank angle can give rise to the large depth of the surface metamorphic layer and average cutting force in the nanocutting process, which is consistent with previous work [10,38].

### 3.4. Effect of the Round Edge Diameter

**Figure 11:**
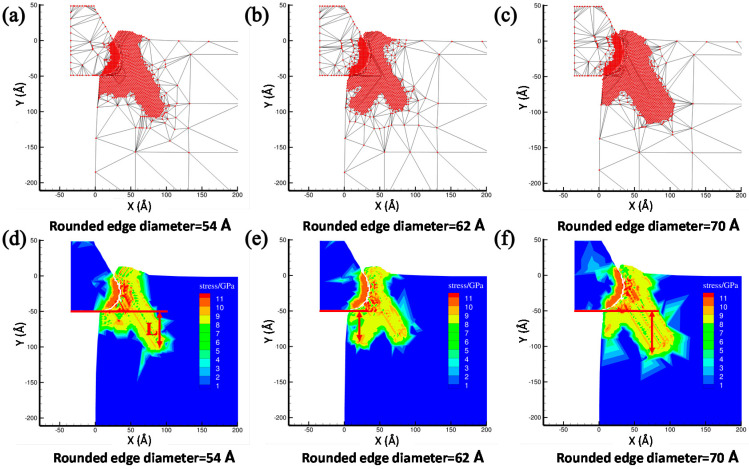
The atomic displacement and corresponding stress distribution at the 200th load step with varying the tool rounded edge diameter from 54 to 70 Å. (**a**) The atomic displacement when the rounded edge diameter= 54 Å; (**b**) the atomic displacement when the rounded edge diameter = 62 Å; (**c**) the atomic displacement when the rounded edge diameter = 70 Å; (**d**) the corresponding stress distribution when the rounded edge diameter = 54 Å; (**e**) the corresponding stress distribution when the rounded edge diameter = 62 Å and (**f**) the corresponding stress distribution when the rounded edge diameter = 70 Å.

**Figure 12:**
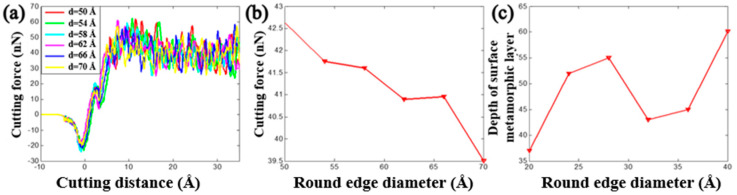
The cutting force and depth of surface metamorphic layer obtained from QC by varying the tool rounded edge diameter. (**a**) Cutting force vs. the cutting distance under different tool rounded edge diameter d; (**b**) evolution of the average cutting force when varying the tool rounded edge diameter and (**c**) evolution of the depth of the surface metamorphic layer when varying the tool rounded edge diameter d.

**Figure 13:**
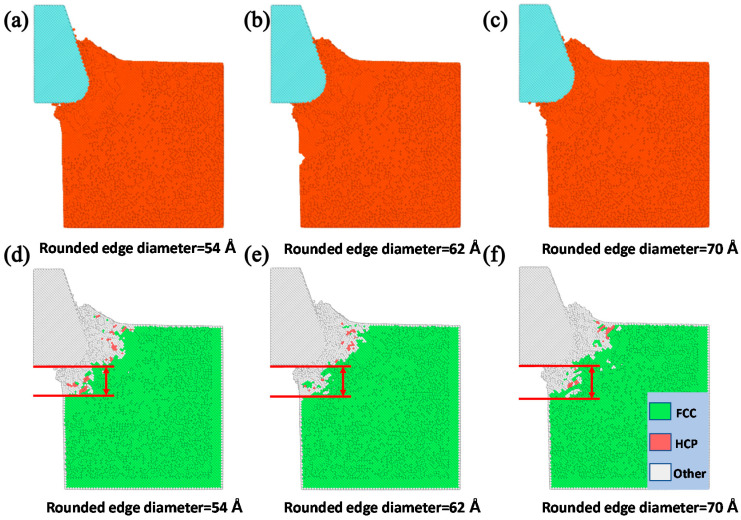
The atomic displacement and lattice distribution in the MD simulation at the 200th load step with varying the tool rounded edge diameter. (**a**) The atomic displacement when the rounded edge diameter = 54 Å; (**b**) the atomic displacement when the rounded edge diameter = 62 Å; (**c**) the atomic displacement when the rounded edge diameter = 70 Å; (**d**) the lattice distribution when the rounded edge diameter = 54 Å; (**e**) the lattice distribution when the rounded edge diameter = 62 Å and (**f**) the lattice distribution when the rounded edge diameter = 70 Å.

**Figure 14:**
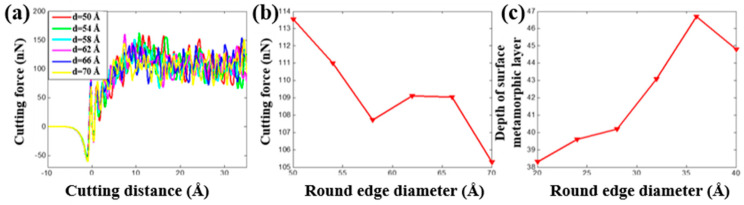
The cutting force and depth of the surface metamorphic layer obtained from MD by varying the tool rounded edge diameter. (**a**) Cutting force vs. the cutting distance under a different tool rounded edge diameter d; (**b**) evolution of the average cutting force when varying the tool rounded edge diameter and (**c**) evolution of the depth of the surface metamorphic layer when varying the tool rounded edge diameter d.

The tool round edge diameter that is another important tool parameter playing an important role on the nanocutting process. By fixing the initial back-engagement as 50 Å and tool rake angle as 30°, Figure 11a–c illustrates the atomic displacement variation during the cutting process of the copper sample at the 200th step by varying the tool round edge diameter from 50 to 70 Å. It can be found that the chip thickness decreased gradually, the deformation region of the lattices expanded gradually and the FE region of the cutting tool close to the contact region was refined automatically with the increment of the rounded edge diameter of the tool, which might be affected by the increment of the contact area between the tool and the workpiece. Correspondingly, the internal stress distribution of the workpiece is illustrated in Figure 11d–f. It can be clearly observed that the stress mainly concentrates in the contact region and expands into the workpiece gradually with the increment of the tool round edge diameter. Additionally, the internal stress of the cutting tool expanded gradually from the tool rounded edge to the interior. The depth of the surface metamorphic layer exhibited an increasing trend with the increment of the tool rounded edge diameter. Figure 12a illustrates the variation of cutting force with respect to the cutting distance under a different tool rounded edge diameter. It can be found that the cutting force initially increased drastically with the increment of cutting distance, and then gradually stabilized, which was identical to the effect of the above-mentioned cutting parameters (as shown in Figure 5a and Figure 8a). Figure 12b illustrates the average cutting force from 25 to 35 Å cutting distance decreased slightly from 42.6 to 39.5 nN with the increment of the tool rounded edge diameter, which resulted from the interfacial interaction between the tool rounded edge and the workpiece. Additionally, the results obtained by MD simulation are shown in Figure 13 and Figure 14, indicating that the average cutting force decreased and the depth of HCP lattice increased gradually with the increment of tool rounded edge diameter, which was comparable to the above results obtained by QC method. We came to the conclusion that the small tool round edge diameter leads to the increment of depth of the surface metamorphic layer and the decrement of the average cutting force in the nanocutting process, which is in correspondance with previous work [27]. 

## 4. Conclusions

In this work, we proposed a modified QC method by introducing the Cauchy local stress and the material removal strategy to simulate the nanocutting process as close as possible to practice. Compared with MD and FE methods, the QC method integrates the finite element theory into the molecular calculation, and thus can balance the simulation accuracy and the computation, which extends the MD method to a larger scale scope, especially for the large-scale precise workpiece. To verify the effectiveness and applicability of the modified QC method in the nanocutting process, a series of numerical simulations about the nanocutting process of copper sample were implemented. We assessed the effects of the cutting tool parameters and back-engagement on the cutting force, stress distribution and the depth of the surface metamorphic layer during the nanocutting process of the copper sample. The results were comparable to those from the MD method, which indicate the effectiveness and applicability of the modified QC method in the nanocutting process. However, QC is still a two-dimensional simulation method in the work, which has to make a compromise on the simulation accuracy. Further work will mainly focus on the three-dimensional QC method. All in all, our work might provide an applicable and efficient strategy to investigate the nanocutting machining mechanism of the large-scale workpiece and shed light on its applications in super-precise and high-quality devices.

## Supplementary Materials

The following are available online at https://www.mdpi.com/1996-1944/13/14/3135/s1, Figure S1: The comparison between the Cauchy stress and Kirchoff stress in the nanoindentation simulation of single crystal copper, Figure S2: The effect of the material removal strategy on the nano-cutting process, Table S1: The detailed parameters of the EAM potentials of single crystal copper.
