# Supplementary material for: Multiscale Assessment of Nanoscale Manufacturing Process on the Freeform Copper Surface
Source: Materials (Basel). 2020 Jul 14;13(14):3135. doi: 10.3390/ma13143135 (PMC7412037; doi:10.3390/ma13143135)
Supplement: Supplementary file 1 [file materials-13-03135-s001.pdf]

materials

Supplementary materials

Multiscale Assessment of Nanoscale Manufacturing Process on the Freeform Copper Surface

Yafei Xu [1], Handing Liu [1], Liuyang Zhang [1,2,*] and Matthew Becton [3]

[1] State Key Laboratory for Manufacturing Systems Engineering, Jiaotong University, Xi'an 710049, China; xyf2492229210@stu.xjtu.edu.cn (Y.X.); lhd.1992.lc@stu.xjtu.edu.cn (H.L.)
[2] Xi'an Jiaotong University Shenzhen Academy, Nanshan District, Science and Technology Park, Shenzhen 518057, China
[3] College of Engineering, University of Georgia, Athens, GA 30602, USA; becton@uga.edu
* Corresponding author: liuyangzhang@xjtu.edu.cn

Supplementary Tables

Table S1. The detailed parameters of the EAM potentials of single crystal copper [1].

Parameters	Values	Parameters	Values	Parameters	Values
r_e	2.556	A	0.328	B	0.469
α	7.670	β	4.091	κ	0.431
λ	0.863	m	20	n	20

Supplementary Figures

The comparison between the Kirchoff stress and Cauchy stress

To elaborate the difference between the Kirchoff stress and Cauchy stress for nano-cutting process, the nanoindentation simulation of the single crystal copper is carried out to validate the effectiveness and applicability of both methods in the local stress calculation in the deformation core region. In the simulation process, the constant downward pressure is imposed on the surface of single crystal copper with the size 200 nm × 100 nm by the nanoindenter of 1 nm length. **Error! Reference source not found.**a,b illustrates the Cauchy stress distribution and Kirchoff stress distribution during the nanoindentation, respectively. It can be found that the Kirchoff stress is lower in the deformation core region than other deformation region, which is contrary to the results in previous literatures [2]. However, the Cauchy stress distribution mainly concentrates on the deformation core region which is consistent with the practical stress condition during the nanoindentation. Additionally, the calculated max Cauchy stress is approximately identical to the theoretical analysis under different initial pressure, which indicates that the Cauchy stress is more suitable for the characterization of stress distribution in the deformation core region during the nano-cutting process.

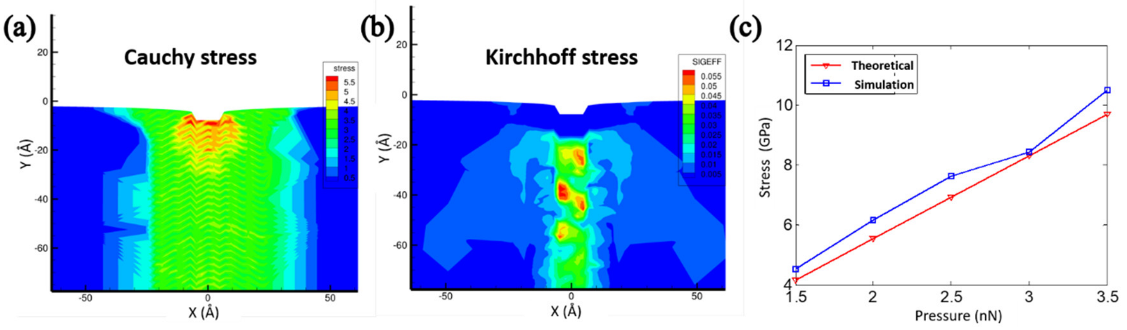

Figure S1. The comparison between the Cauchy stress and Kirchoff stress in the nanoindentation simulation of single crystal copper. (**a**) The Cauchy stress distribution; (**b**) The Kirchoff stress distribution; (**c**) The comparison of the Cauchy stress between the simulation results and the theoretical analysis under different initial pressure.

The material removal strategy

To unveil the effect of the material removal strategy on the nano-cutting process, a series of nano-cutting simulations of single crystal copper are carried out with the initial back engagement 40 Å, tool rank angle 30° and rounded edge diameter 60 Å. **Error! Reference source not found.**a,b illustrates the atomic displacement at 200th load step obtained by the original QC program and the modified QC program during cutting process, respectively. It can be found that the unreasonable dislocation band of 157 Å depth that exceeds the criteria limit appears due to the accumulation of excessive distorted lattice during the cutting process by using the original QC method (as shown in **Error! Reference source not found.**a), which is inconsistent with the results obtained by the MD method (as shown in **Error! Reference source not found.**c). However, the reasonable dislocation band of 55 Å depth appears due to removal of the excessive distorted lattice caused by material removal effect of the modified QC method (as shown in **Error! Reference source not found.**b), which is consistent with the results obtained by the MD method (as shown in **Error! Reference source not found.**c). Therefore, the modified QC method with material removal can effectively fulfill the investigation on the nano-cutting process of single crystal copper.

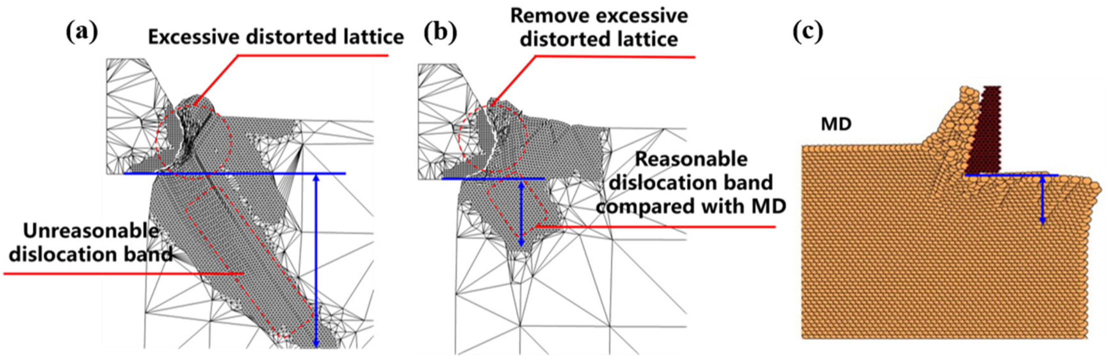

Figure S2. The effect of the material removal strategy on the nano-cutting process. (**a**) The atomic displacement at 200th load step obtained by using the original QC program; (**b**) The atomic displacement at 200th load step obtained by using the modified QC program; (**c**) The atomic displacement at 200th load step obtained by using MD method.

Reference

1. Hong, R.T.; Huang, M.J.; Yang, J.Y. Molecular dynamics study of copper trench filling in damascene process. *Mater. Sci. Semicond. Process.* **2005**, *8*, 587–601.
2. Zhu, A.; He, D.; He, R.; Zou, C. Nanoindentation simulation on single crystal copper by quasi-continuum method. *Mater. Sci. Eng. A* **2016**, *674*, 76–81.
